# Spatial and temporal patterns of diarrhoea in Bhutan 2003–2013

**DOI:** 10.1186/s12879-017-2611-6

**Published:** 2017-07-21

**Authors:** Kinley Wangdi, Archie CA Clements

**Affiliations:** 1 Department of Global Health, Research School of Population Health, College of Medicine, Environment and Biology, The Australian National University, Canberra, Australia; 2 Phuentsholing General Hospital, Phuentsholing, Bhutan

**Keywords:** Time series analysis, Spatial analysis, Bayesian analysis, Diarrhoea, Bhutan

## Abstract

**Background:**

To describe spatiotemporal patterns of diarrhoea in Bhutan, and quantify the association between climatic factors and the distribution and dynamics of the disease.

**Methods:**

Nationwide data on diarrhoea were obtained for 2003 to 2013 from the Health Information and Management System (HIMS), Ministry of Health, Bhutan. Climatic variables were obtained from the Department of Hydro Met Services, Ministry of Economic Affairs, Bhutan. Seasonal trend decomposition was used to examine secular trends and seasonal patterns of diarrhoea. A Bayesian conditional autoregressive (CAR) model was used to quantify the relationship between monthly diarrhoea, maximum temperature, rainfall, age and gender.

**Results:**

The monthly average diarrhoea incidence was highly seasonal. Diarrhoea incidence increased by 0.6% (95% CrI: 0.5–0.6%) for every degree increase in maximum temperature; and 5% (95 Cr I: 4.9–5.1%) for a 1 mm increase in rainfall. Children aged <5 years were found to be 74.2% (95% CrI: 74.1–74.4) more likely to experience diarrhoea than children and adults aged ≥5 years and females were 4.9% (95% CrI: 4.4–5.3%) less likely to suffer from diarrhoea as compared to males. Significant residual spatial clustering was found after accounting for climate and demographic variables.

**Conclusions:**

Diarrhoea incidence was highly seasonal, with positive associations with maximum temperature and rainfall and negative associations with age and being female. This calls for public health actions to reduce future risks of climate change with great consideration of local climatic conditions. In addition, protection of <5 years children should be prioritize through provision of rotavirus vaccination, safe and clean drinking water, and proper latrines.

**Electronic supplementary material:**

The online version of this article (doi:10.1186/s12879-017-2611-6) contains supplementary material, which is available to authorized users.

## Background

Diarrhoea is associated with high childhood morbidity and mortality [1, 2] and claims two million lives each year in developing countries [3]. It is the second leading cause of death in children <5 years old, and World Health Organization (WHO) estimates around 760,000 children <5 years die each year from this disease [4]. About 88% of diarrhoea-associated deaths are attributable to unsafe water, inadequate sanitation, and insufficient hygiene [5, 6].

Unsafe water can be associated with the changes in metrological factors such as flooding, temperature, humidity, and rainfall [7, 8]. The increased water level as a result of annual flooding can contaminate water sources by transporting pathogens [9], triggering waterborne diseases including diarrheal outbreaks [10, 11]. Diarrhoea has been reported to demonstrate strong seasonal variation related to climate [12–14]. Increased diarrheal disease reputedly associated with climate change has been reported [15, 16]. In places with water scarcity, use of rain water as a source of domestic and drinking water might contribute to diarrhoea occurrence as a result of contamination by human and industrial wastes [17–20].

Diarrhoeal disease continues to cause significant morbidity and mortality in Bhutan. Diarrhoea was ranked amongst the top ten diseases in terms of number of cases in the last 5 years [21–23]. In 2015, diarrhoea was the fifth common conditions with 51,593 reported cases [23]. The high burden of diarrhoea occurs despite increases in sanitation with increased coverage of latrines and drinking water through rural water supply schemes (RWSS). In 2000, national coverage of sanitary latrines and piped water was 87 and 65%, respectively [24]; whereas by 2011, coverage of sanitary latrines and safe drinking water increased to 91 and 83%, respectively [25].

While the patterns of the climate–diarrhoea relationship have been described elsewhere in the world [11, 26], no investigation of such a relationship has been carried out in Bhutan. Additionally, no previous studies have been undertaken using spatiotemporal modelling approaches to identify spatial and temporal clusters of diarrhoea in Bhutan. Therefore, this paper aimed to: (i) describe seasonal patterns and temporal trends in diarrhoea, and (ii) identify local clusters of diarrhoea at district level and determine the association between climatic factors such as maximum temperature and rainfall and the spatiotemporal distribution of the disease.

## Methods

### Data source

In this study, nationwide data on diarrhoeal disease were obtained for 2003 to 2013 from the Health Information and Management System (HIMS), Ministry of Health, Bhutan. These data contain the diarrhoea cases reported by health centers through district health offices to the HIMS. The reported data were aggregated by age (<5 years or ≥5 years) and gender. Population estimates used in this study were from the National Statistical Bureau (NSB) and the Office of the Census Commissioner of Bhutan [27, 28]. Climatic variables (temperature and rainfall) for respective districts were obtained from the Department of Hydro Met Services under the Ministry of Economic Affairs, Bhutan.

### Exploration of seasonal patterns and temporal trends

The average monthly diarrhoea incidence, rainfall and temperature were calculated from the full time-series. These were plotted to show temporal patterns in diarrhoea and weather variables. The time series of diarrhoea incidence was decomposed using seasonal-trend decomposition based on locally weighted regression to show: the seasonal pattern, the temporal trend and the residual variability. This method uses loess smoothing on sub-series of each season (i.e. month in this case) separately to estimate the seasonal pattern. The seasonal component is then removed from the time series prior to further smoothing to estimate the trend, leaving the residual values.

### Data analysis

Initially, a preliminary Poisson regression of diarrhoea cases was undertaken to select covariates. Two climatic variables were entered as covariates, namely maximum temperature and rainfall, without a lag, and with 1 month and 2 month lag times. The covariates from the model with the lowest Akaike’s information criterion (AIC) and Bayesian information criterion (BIC) were included in the final analysis.

Separate Poisson regression models were constructed in a Bayesian framework using the WinBUGS software, version 1.4.3 (MRC Biostatistics Unit 2008). The first model (Model I), assumed that spatial autocorrelation was not present in the relative risk of diarrhoea. This model was developed including temperature, rainfall, age (<5 and ≥5 years) and gender as explanatory variables, and unstructured random effect for districts; the second model (Model II) included the same explanatory variables and a spatially structured random effect; the final model (Model III), a convolution model, contained all of the components of the preceding two models.

The last model assumed that the observed counts of diarrhoea, *Y*, for *i*th district (*i* = 1..20) in the *j*th month (January 2003–December 2013) followed a Poisson distribution with mean (μ_ij_), that is,

$$ \begin{array}{c}\kern1em {Y}_{ij}\sim \mathrm{Poisson}\left({\upmu}_{ij}\right)\kern1em \\ {}\kern1em  \log \left({\upmu}_{ij}\right)= \log \left({\mathrm{E}}_{ij}\right)+{\theta}_{ij}\kern1em \\ {}\kern1em {\theta}_{ij}=\alpha +{\beta}_1\times {\mathrm{Tempmax}}_{ij}+{\beta}_2\times {\mathrm{Rainfall}}_{ij}+{\beta}_3\times \mathrm{Age}+{\beta}_4\times \mathrm{Sex}+{\mathrm{u}}_i+{\mathrm{s}}_i\kern1em \end{array} $$

where E_*ij*_ is the expected number of cases in district *i*, month *j* (acting as an offset to control for population size) and θ_*ij*_ is the mean log relative risk (RR); α is the intercept, and *β*_*1*_ *, β*_*2*_ *, β*_*3*_ *,* and *β*_*4*_ the coefficients for maximum temperature, rainfall, age and sex, respectively; u_*i*_ is the unstructured random effect with mean zero and variance σ_u_^2^ and s_*i*_ is the spatially structured random effect with mean zero and variance σ_s_^2^.

A conditional autoregressive (CAR) prior structure was used to model the spatially structured random effect. Spatial relationships between the districts were determined using an adjacency weights matrix. For two districts sharing a border, a weight of 1 was assigned, while for pairs of districts not sharing a border, a weight of 0 was assigned. A flat prior distribution was specified for the intercept, whereas a non-informative normal prior distribution was used for the coefficients. The priors for the precision of unstructured and spatially structured random effects were specified using non-informative gamma distributions with shape and scale parameters equal to 0.01.

An initial burn-in of 1000 iterations was run, and these iterations were discarded. Subsequent blocks of 20,000 iterations were run and examined for convergence. Convergence was assessed by visual inspection of posterior density and history plots, and occurred at approximately 100,000 iterations for each model. Ten thousand values from the posterior distributions of each model parameters were stored and summarised for the analysis (posterior mean and 95% credible intervals [CrI]). The deviance information criterion (DIC) was calculated for model selection, where a lower DIC indicates a better model fit. In all analyses, an α-level of 0.05 was adopted to indicate statistical significance (as indicated by 95% CrI for relative risks (RR) that excluded 1).

Seasonality decomposition was carried out using the R statistical package, release 3.3.1. ArcMap software was used to generate the maps of spatial distribution of posterior means of the unstructured and structured random effects obtained from the three models.

## Results

### Exploration of seasonal patterns and temporal trends

**Fig. 1:**
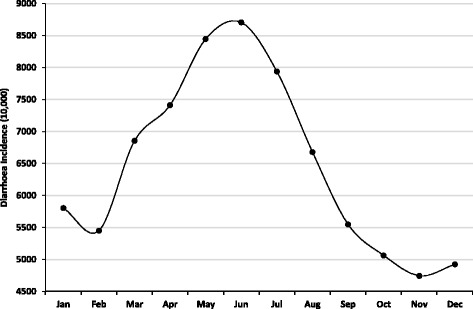
Monthly average diarrhoea incidence rates per 10,000 population

A total of 1,483,316 diarrhoea cases were notified to HMIS by different health centres of Bhutan from 1 January 2003 to 31 December 2013. The cases fluctuated during the study period, with the highest number of cases reported in 2009 followed by 2013 with 140,703 and 139,666 cases, respectively. Lowest numbers of cases were recorded in 2010 followed by 2011 with 129,369 and 129, 670 cases. In both sex less than 40% of cases were in children (<5 years). The monthly average diarrhoea incidence during the study period showed two peaks, with a small peak in January and a much larger peak in June. There was a lower incidence towards the end of the year from September–December, with the lowest reported incidence in November (Fig. 1).

**Fig. 2:**
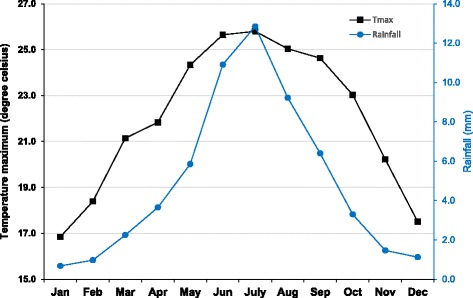
Monthly averages of rainfall (*blue*) and maximum temperature (*black*)

**Fig. 3:**
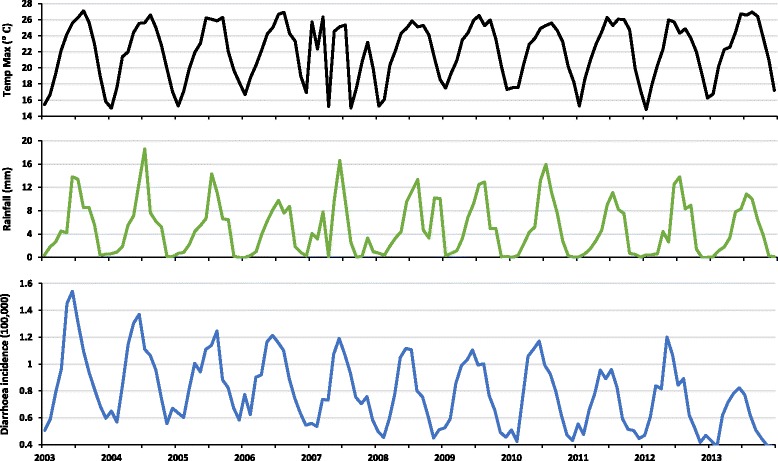
Time series mapping of temperature (maximum) in *black*, rainfall in *green* and diarrhoea per 100,000 in *blue*

The seasonal patterns of rainfall, and maximum temperature showed warmer, wetter months in the middle of the year (June and July) and cooler, dryer months traversing the end of the year (November to February). The two hottest years were 2003 and 2013, while two wettest years were 2004 and 2007. The lowest rainfall was recorded in 2006 and 2013 respectively (Figs. 2 and 3).

**Fig. 4:**
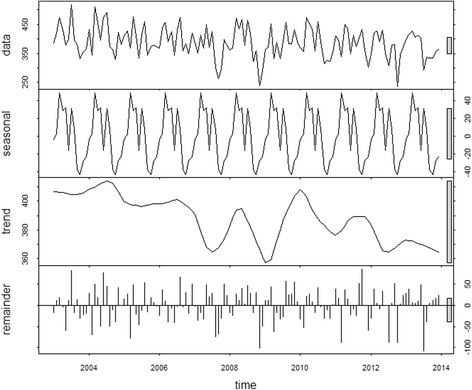
Decomposed total diarrhoea time-series. Data presented as incidence rates per 10,000 population

**Fig. 5:**
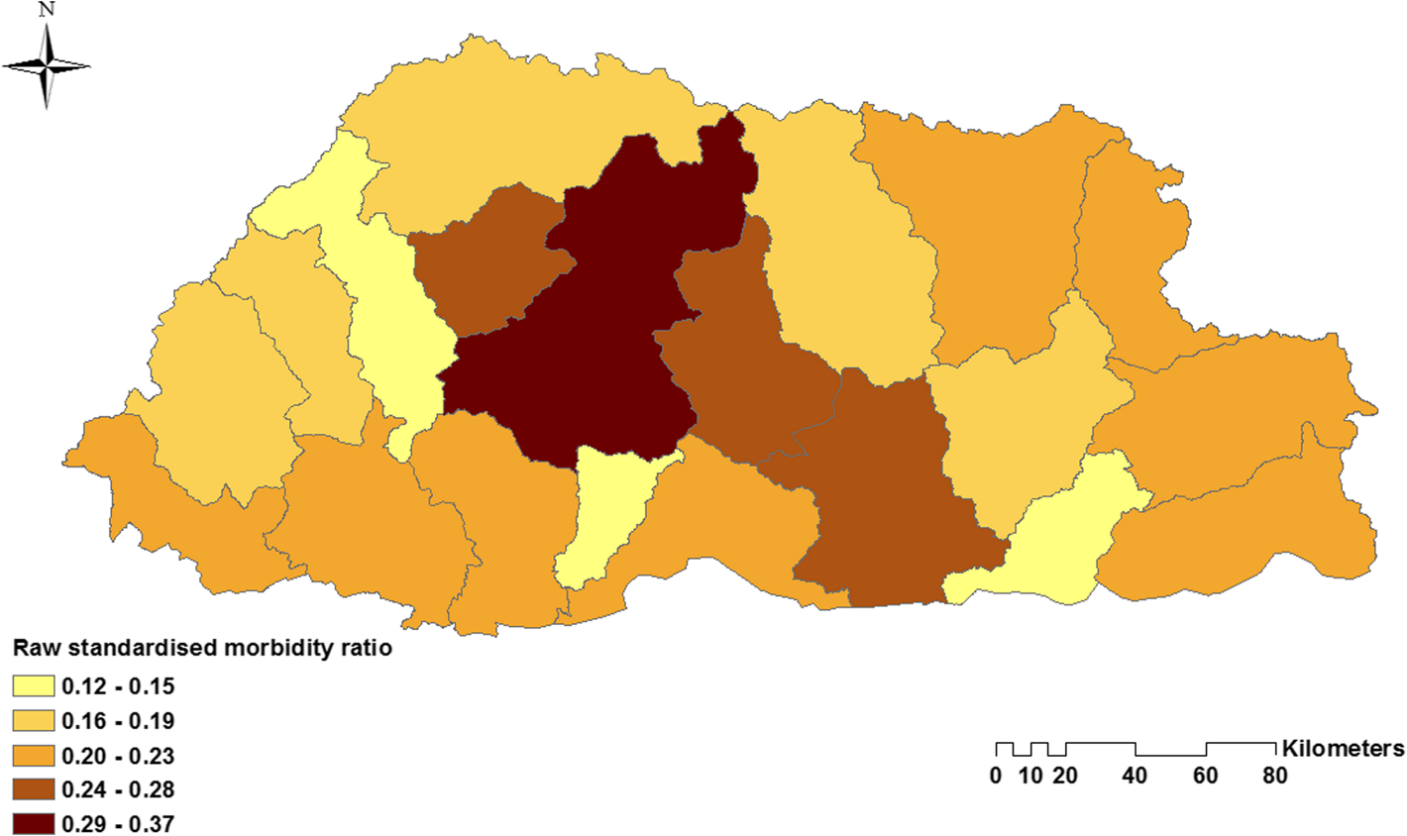
Raw standardised morbidity ratios for diarrhoea by district of Bhutan for the study period 2003–13

The time-series decompositions of the diarrhoea case numbers show clear seasonal patterns with the major peak in June and a declining trend until 2013, but with a resurgence in 2014 (Fig. 4)**.** The map of standardised morbidity ratios (SMR) by districts show that higher cases of diarrhoea is observed in the central part of Bhutan (Fig. 5).

### Association between diarrhoea and climatic factors

Models containing the climatic factors (maximum temperature and total rainfall) without a lag had the best fit, with lower AIC and BIC. Both maximum temperature and total rainfall for each district were significantly (*p <* 0.0001) associated with the incidence of diarrhoea in the preliminary models (Table 1).

**Table 1:** Covariate effects from preliminary models of diarrhoea incidence, Bhutan, 2003–13

Climatic variables	RR	95% CI	*P* value	AIC	BIC
No lag					
Temp max	0.996	0.995, 0.996	<0.0001	184,006.6	184,024.2
Rainfall	0.998	0.997, 0.998	<0.0001		
Lag 1 month					
Temp max	0.996	0.995, 0.996	<0.0001	184,760.8	184,778.4
Rainfall	0.998	0.998, 0.999	<0.0001		
Lag 2 months					
Temp max	0.998	0.997, 0.998	<0.0001	184,555.9	184,573.5
Rainfall	0.998	0.998, 0.998	<0.0001		

### Spatio-temporal model

**Fig. 6:**
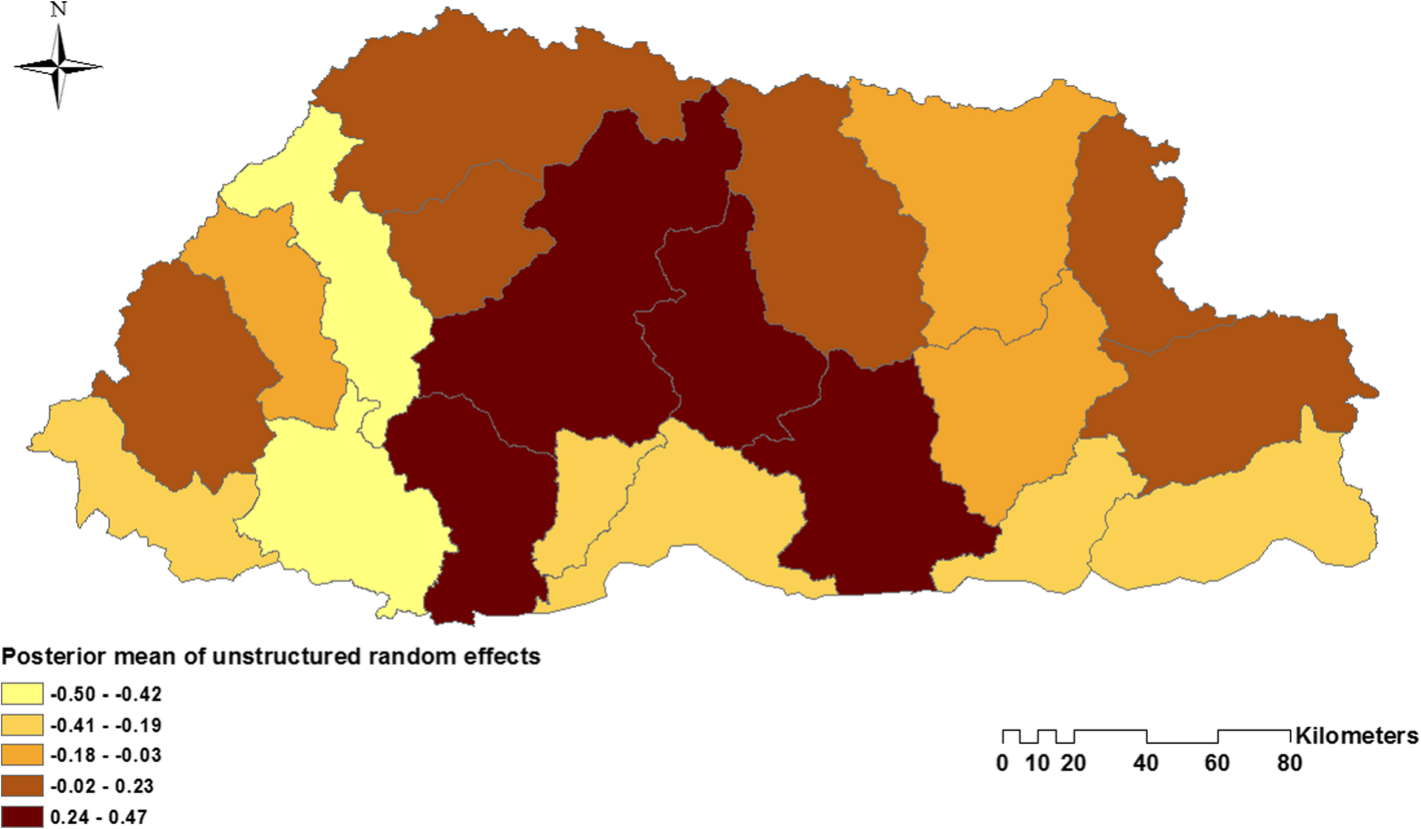
Spatial distribution of the posterior means of unstructured random effects for diarrhoea in Bhutan in Model I

When the three models were compared using the DIC, Model I containing unstructured random effects had the best fit. In Model I, there was found to be an increase in diarrhoea cases by 0.6% (95% CrI: 0.5–0.6%) for every degree increase in maximum temperature; and an increase in diarrhoea cases of 5% (95 Cr I: 4.9–5.1%) for a 1 mm increase in rainfall. Children aged <5 years were found to be 74.2% (95% CrI: 74.1–74.4) more likely to experience diarrhoea than children and adults aged ≥5 years; and females were found to be 4.9% (95% CrI: 4.4–5.3%) less likely to suffer from diarrhoea as compared to males (Table 2). The maps of the posterior means of the spatially unstructured random effects demonstrated little evidence of spatial clustering after accounting for the model covariates (Fig. 6, Additional file 1: Figure S1 and Additional file 2: Figure S2).

**Table 2:** Regression coefficients, RRs and 95% CrI from Bayesian spatial and non-spatial models of diarrhoea incidence, Bhutan, 2003–13

Model/variables	Coefficient, posterior mean (95% CrI)	RR, posterior mean (95% CrI)
Model I (Unstructured)		
α (Intercept)	−0.034 (−0.03, 0.143)	
Temperature Maximum (°C)^a^	0.006 (0.005, 0.006)	1.006 (1.005, 1.006)
Rainfall (mm)^a^	0.0489 (0.048, 0.049)	1.050 (1.049, 1.051)
Age^b^	−1.356 (−1.361, −1.351)	0.258 (0.256, 0.259)
Sex	−0.05 (−0.055, −0.045)	0.951 (0.947, 0.956)
Heterogeneity		
Structured	-	-
Unstructured	0.073 (0.174, 0.044)	-
DIC	210,591	
Model II (Structured)		
α (Intercept)	−0.012 (−0.028, 0.004)	
Temperature Maximum (°C)^a^	0.006 (0.005, 0.006)	1.006 (1.005, 1.006)
Rainfall (mm)^a^	0.0489 (0.048, 0.049)	1.050 (1.049, 1.051)
Age^b^	−1.356 (−1.361, −1.351)	0.258 (0.256, 0.259)
Sex	−0.05 (−0.055, −0.045)	0.951 (0.947, 0.956)
Heterogeneity	-	
Structured	0.218 (0.87, 0.245)	-
Unstructured	-	-
DIC	210,594	
Model III (Structured and unstructured)		
α (Intercept)	−0.032 (−0.161, 0.086)	
Temperature Maximum (°C)^a^	0.006 (0.005, 0.006)	1.006 (1.005, 1.006)
Rainfall (mm)^a^	0.049 (0.048, 0.049)	1.050 (1.049, 1.051)
Age^b^	−1.356 (−1.361, −1.351)	0.258 (0.256, 0.259)
Sex	−0.05 (−0.055, −0.045)	0.951 (0.947, 0.956)
Heterogeneity		
Structured	0.067 (0.151, 0.033)	-
Unstructured	0.007 (0.157, 0.038)	-
DIC	210,598	

^a^District specific

^b^Age categorized as <5 years and ≥5 years

## Discussion

This is the first study to explore spatiotemporal patterns of diarrhoea in Bhutan at the national level. The findings from this study showed clear seasonal patterns in diarrhoeal incidence with two peaks every year, December–January and the largest peak occurring in June, at the same time that temperature and rainfall demonstrate seasonal peaks. A study in the capital city Thimphu showed that rotavirus infection was highest during the winter-spring (December–April) season, while diarrhoea was highest during the summer (May–July) [29]. Similar significant peaks of rotavirus were observed during the cold months in India [30].

There was positive association between climatic variable and diarrhoea cases and a negative association with increasing age and being female. There was no evidence of spatial clustering of diarrhoea risk after accounting for the covariates, suggesting that variability in rainfall and temperature explain much of the spatiotemporal dynamics of the disease.

The use of time-series analysis can provide valuable insights into the seasonal patterns of diarrhoea transmission. The seasonality in the annual incidence of diarrhoeal disease in Bhutan supports the role of climatic drivers, as have been implicated in other countries [31–33]. The positive relationships between diarrhoea and temperature and rainfall in our present study is broadly consistent with previous studies in Japan, China, Peru, Fiji, and Bangladesh [15, 18, 34–37]. The WHO and the Intergovernmental Panel on Climate Change (IPCC) have identified changes in the incidence of diarrhoea as one of the most important future health effects of climate change [38].

The positive association between temperature and diarrhoea is biologically plausible given that higher temperatures promote the growth of bacteria, although some enteric viruses have been suggested to increase survival and transmission under lower temperatures [39]. Increased temperatures could lead to food poisoning because food spoils easily in warmer weather [40]. Higher temperature can also lead to dietary and hygiene variations with increased demand for water, water shortages and sub-optimal sanitation, which could facilitate transmission of bacteria and other pathogens.

Diarrhoea cases increased with the amount of rainfall in our study. There are a number of plausible explanation for this occurrence; firstly, the main source of drinking water in Bhutan is surface water from streams. Increased rainfall may affect levels of contaminates in the drinking water as had been reported elsewhere [17, 20, 37, 41]. This could be true for Bhutan because most water from the rural water supply scheme (RWSS) is not chemically treated as opposed to water in urban settings. Secondly, increased rainfall can cause flooding and changes in living environment. Floodwaters can foster the growth of many pathogens and lead to contamination of the food supply. Importantly, flood water can incapacitate the sewage system and increase transmission of diarrhoeal pathogens [42–44].

The risk of diarrhoea cases was lower in people aged ≥5 years in this study confirming diarrhoea is primarily a disease of children [45–48]. Rotavirus is the most common cause of diarrhoea among children <5 years of age globally [49, 50]. The underlying pathogens for diarrhoea was not reported in this study. However, a study in Thimphu district of Bhutan reported 22.3% of the stools were positive for rotavirus in children <5 years of age with diarrhoea [29]. Immunization against rotavirus in not routinely administered in children in Bhutan. Introducing immunization against rotavirus is likely to significantly reduce childhood diarrhoea. Other studies have reported significant reductions in both diarrhoea deaths and diarrhoea-related hospital admissions following rotavirus vaccination [51, 52]. As in many other studies, females were found to be less likely than males to suffer from diarrhoea [53, 54], although this has not always been found to be the case elsewhere [55]. The risk of diarrhea in females being greater than males could be as a result of possible gender inequalities in health-care seeking and differential in the care of female children [56–59]. In addition, females are at a higher risk of other medical conditions such as irritable bowel syndrome that could lead to diarrhoea [60, 61].

Whilst no residual spatial clustering was identified, high-incidence districts were Dagana, Trongsa, Wangdue, and Zhemgang. The levels of sanitation and access to safe drinking water in these districts were reported to be amongst the lowest in Bhutan [25] and poverty indicators for these districts are among the worst in the country [62]. Poverty and sanitation are an important determinants of diarrhoea risk [63, 64]. It would be worthwhile to explore these variables in models in the future, if there variables become available.

There are several limitations in this study. First, diarrhoea cases were not all laboratory confirmed. Second, age could not be stratified as <1 year and 1-4 years because surveillance data from 2003 to 2009 aggregated all diarrhea cases <5 years into one group. Third, people with mild forms of diarrhoea may not seek medical care and remain unreported and access to health care may vary across districts leading to differences in reporting of diarrhoea – the impact of reporting bias on observed spatiotemporal patterns is difficult to assess. Fourth, the underlying pathogens were not reported, which prevented examination of the dynamics of specific pathogens. Of note, other explanations for seasonal variation in diarrhoea also need to be considered, such as temporal oscillations in host susceptibility due to changes in neuroendocrine function and immune response [65].

## Conclusion

In conclusion, incidence of diarrhoea in Bhutan was highly seasonal and strongly associated with local weather factors, including temperature and rainfall. This calls for public health actions to reduce future risks of climate change with great consideration of local climatic conditions. In addition, protection of <5 years children should be prioritize through provision of rotavirus vaccination, safe and clean drinking water, and proper latrines.

## Additional files

Additional file 1: Figure S1. Spatial distribution of the posterior means of structured random effects for diarrhoea in Bhutan in Model II. (TIFF 2238 kb)

Additional file 2: Figure S2. Spatial distribution of the posterior means of random effects for diarrhoea in Bhutan in Model III. (a) Spatially unstructured random effects (b) structured random effects. (TIFF 2610 kb)

## Abbreviations

AIC: Lowest Akaike’s information criterion
BIC: Bayesian information criterion
CAR: Conditional autoregressive
CrI: Credible intervals
DIC: Deviance information criterion
HIMS: Health information and management system
IPCC: Intergovernmental panel on climate change
NSB: National Statistical Bureau
RR: Relative risk
RWSS: Rural water supply schemes
SMR: Standardised morbidity ratios
WHO: World Health Organization
