# Supplementary figures and images for: Spatial and temporal patterns of diarrhoea in Bhutan 2003–2013
Source: BMC Infect Dis. 2017 Jul 21;17:507. doi: 10.1186/s12879-017-2611-6 (PMC5521140; doi:10.1186/s12879-017-2611-6)

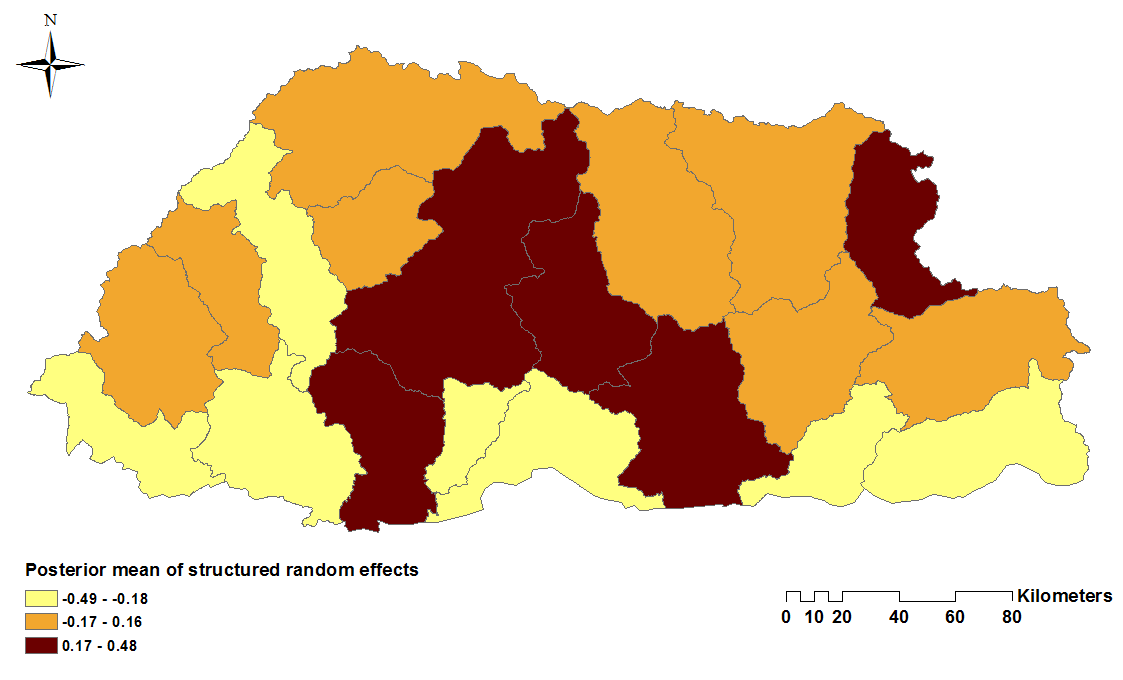

Supplement: Supplementary file 1 — Spatial distribution of the posterior means of structured random effects for diarrhoea in Bhutan in Model II. (TIFF 2238 kb) [file 12879_2017_2611_MOESM1_ESM.tif]

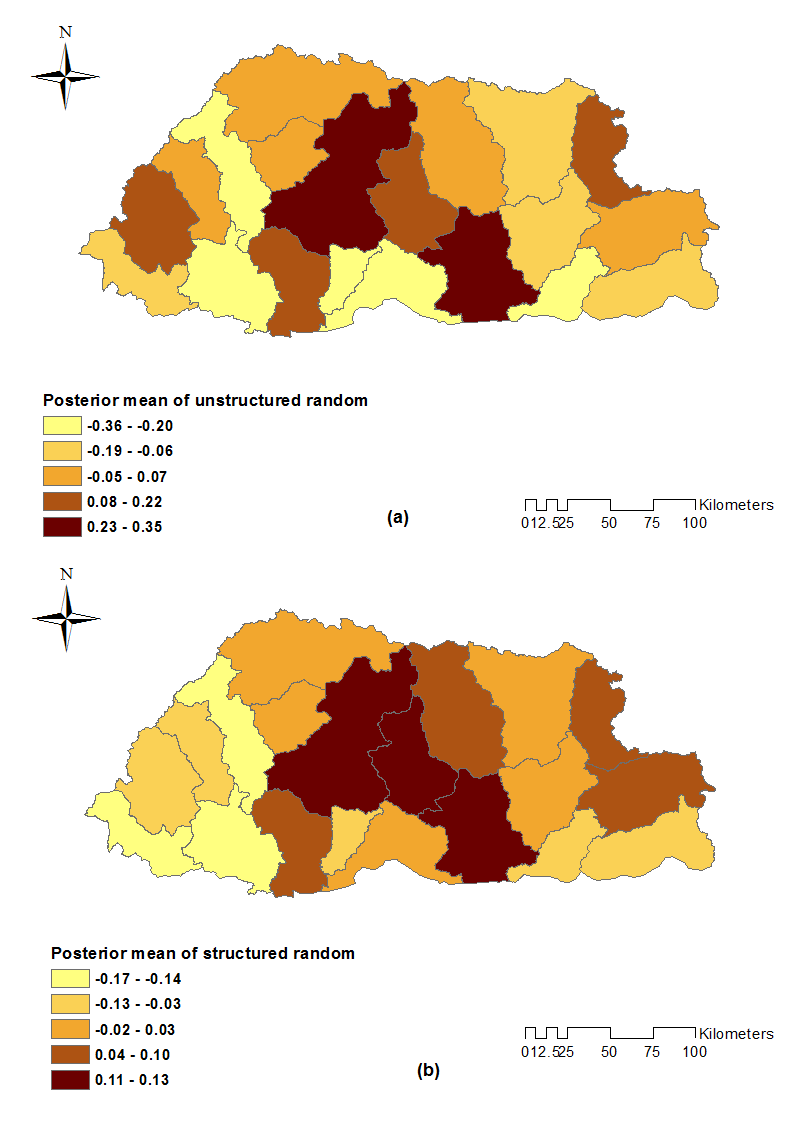

Supplement: Supplementary file 2 — Spatial distribution of the posterior means of random effects for diarrhoea in Bhutan in Model III. (a) Spatially unstructured random effects (b) structured random effects. (TIFF 2610 kb) [file 12879_2017_2611_MOESM2_ESM.tif]
